# An Inquiry into the Character of Green and Melænal Discharges from the Bowels

**Published:** 1859-09

**Authors:** S. G. Armor

**Affiliations:** Formerly Professor of the Practice of Medicine in the Medical College of Ohio, and late Professor of Pathology and Clinical Medicine in the Medical Department of the Missouri University


ART. III.—*An Inquiry into the Character of Green and Melænal Discharges from the Bowels.* By S. G. ARMOR, M.D., formerly Professor of the Practice of Medicine in the Medical College of Ohio, and late Professor of Pathology and Clinical Medicine in the Medical Department of the Missouri University.

THE recent labors of Drs. Bernard, Jones, Bidder, Schmidt, Lehmann and others, have added much to our knowledge of the physiological functions of the liver. Indeed, they may be said to have entirely remodeled the whole literature of the subject, and to have invested it with new and peculiar interest.

It is not my purpose, however, at present, to present a *résumé* of the many interesting facts elicited by these investigations, but to attract attention to a single point, and to such facts only as bear upon my present inquiry.

Can we, as has been generally supposed, rely upon green discharges from the bowels as indicating increased or perverted hepatic secretion? Is this the essential practical fact which should chiefly claim our attention? Or must we look to some other pathological condition? And if so, what is that condition?

Whatever views may be entertained relative to these questions, it must be conceded that they cover points of inquiry of great practical importance, for the reason that they relate to a class of diseases of very frequent occurrence.

Of the various secretions poured into the digestive apparatus, none have been regarded with more interest than that of the liver; nevertheless, it is only quite recently that we have arrived at any reliable data as to the real constitution and uses of its secretion. To arrive at these, physiologists have made fistulous openings into the gall-bladder, and, by ligaturing the hepatic duct, have been able to collect and analyze the biliary secretion, and thus to establish many interesting facts, heretofore unknown, as to the true position of this secretion in the organism. I am more particularly interested, however, at present, in noting the effects of the arrest of the hepatic secretion *on the contents of the intestine.* To arrive at this, Drs. Bidder and Schmidt performed the operation of opening a biliary fistula on a great variety of animals under a great variety of circumstances. The details of these experiments are exceedingly minute and accurate. Among other observations of interest to the physiologist, they have demonstrated two facts which have a direct bearing on the subject of my present inquiry: First, that bile undergoes certain alterations in the intestine, "transformations of a catylitic nature, produced by the contact of the bile with the intestinal juices." Second, that it is gradually re-absorbed in its passage from the small to the large intestine; and that finally, in a normal condition of the bowel, it gradually disappears on its road from the liver to the rectum; thus agreeing with Boerhaave, and some of the older physiologists, that "almost the whole mass of the fluid secreted by the liver and poured into the cavity of the intestine, is again secerned or drunk up by the absorbing veins from the alimentary and fæcal contents, before they arrive at the anus."

It has also been demonstrated by experimental observation, that when no bile flows into the intestine, and the animal is fed on *flesh*, rapid decomposition of the contents of the canal occurs, attended by the secretion of fetid gases. When fed on *bread* or starchy substances, the fæces or abdominal gases were odorless, and sour rather than putrid.

In the absence, then, of healthy biliary secretion, we have, it appears, one of two conditions, or the combination of these, depending upon the nature of the food of the animal—*putridity* on one hand, and excessive *acid fermentation* on the other. In either case the color of the fæcal contents is usually changed.

The dark golden-brown color of healthy fæcal dejections depends upon the presence of *biliverdine*—the coloring matter of the bile, which exists in such small quantity that its exact proportion has never been determined.

In cases of biliary obstruction, it is this element of the bile that is re-absorbed into the blood, and stains the tissues and fluids of the body of the peculiar lemon-yellow color which is characteristic of jaundice, the absence of this coloring matter giving rise to light or ash-colored stools.

With these facts before us, let us again submit the point of inquiry: Does the dark-green color of the stools depend, as affirmed by Chomel, and reiterated by many authors and teachers of practical medicine, upon the presence of bile proper? This view, it will be at once perceived, is not in harmony with the facts already referred to; *first*, that biliary substances proper disappear in their passage through the intestine; *second*, that the characteristic color of the fæcal dejections depends not upon the chief constituents of bile, but upon its *coloring* matter, the contents of the bowels becoming stained by its presence just as the tissues and fluids of the body become colored in jaundice.

There has been much speculation as to the character of green stools, *which follow the administration of calomel.* Mercury is administered, and green discharges are regarded as evidence of the secernent action of the drug upon the liver. How far is this opinion founded in fact? It cannot be denied that they frequently *follow* the administration of calomel. It must also be confessed that they are quite *peculiar* to the administration of this drug; and yet it by no means follows, as a logical sequence, that such discharges are evidence of a quickened biliary secretion. What, then, is the explanation of the relation which so frequently exists between the administration of mercury and the green discharges which follow? Dr. Lehmann says, in his Physiological Chemistry, that after calomel has been taken, "we always find mercury in the stools," whether they be green, or black, or the ordinary color. These observations have also been distinctly established by Hermann, Merklin, and others. The sulphide of mercury may be readily separated from the fæcal evacuations, and its chemical nature, when separated, may be easily recognized. "The sulphide of mercury," adds Lehmann, "when finely comminuted, may certainly, like sulphide of iron, give rise to a light-green color with animal substances, and especially with the yellow bile pigment." Indeed, Hermann asserts that powdered calomel, when triturated with yellowish-brown excrements, causes them to assume a greenish color.

Still I would not deny the presence of bile in calomel stools; nor would I deny that the administration of calomel actually causes an increased secretion of bile. The presence of the resinous biliary acids may be readily detected by Pettenkofer's test, especially when the contents of the bowel have been rapidly discharged under the spur of excitement from the action of a mercurial cathartic; and even the green color may depend partly on bile pigment. The question at issue is as to the invariable relation existing between the true constituents of bile and green discharges. Formerly, it will be remembered, this green color was regarded as a sign of the presence of an excess of bile; latterly, however, the necessary presence of bile in green stools has been altogether denied. And the facts certainly clearly indicate that if bile be present in such discharges, it is in diminished rather than in increased quantity.

This subject occupied, for some time before his death, the attention of that eminent analytical chemist, Dr. Golding Bird, whose researches in chemical pathology were always of the most accurate and interesting character. He directed his attention chiefly to the green alvine evacuations of children, "with the view of testing the accuracy of the popular opinion of their being chiefly composed of bile." For this purpose he carefully analyzed a specimen of a green evacuation passed by a hydrocephalic infant while under the influence of mercury. It was a dirty-green, turbid fluid, and by repose separated into three distinct portions. 1. A supernatent oily fluid, presenting a brilliant emerald color. 2. A dense stratum of mucus, coagulated albumen, and epithelial debris. 3. A deposit of large crystals of triple phosphate of magnesia and ammonia in prisms of an apple-green color. The supernatent green fluid was decanted for examination, and its chemical elements carefully analyzed. The following is a view of the results of the examination:—

| | | |
|---|---|---|
| Alcoholic extract, | Organic | 34·50 |
| | Inorganic | 5·50 |
| Aqueous extract, | Organic | 11·25 |
| | Inorganic | 1·75 |
| Insoluble matter, | Organic | 56·00 |
| | Inorganic | 1·00 |
| Water and volatile matter | | 900·00 |
| | | 1000·00 |

The organic portion of the alcoholic extracts, in the foregoing analysis, consisted chiefly of fatty matter, cholesterine, and a green substance supposed to be identical with the so-called *biliverdine*, with but a *mere trace of bile*, barely sufficient to communicate a bitter taste to the extract, but not enough to leave any carbonate of soda in the residue of incineration. The aqueous extract consisted chiefly of ptyalin, with other extractive matters.

An analysis of a green calomel evacuation has been recorded by Simon, with results corresponding with the observations of Dr. Bird.

Professor Kerstein, of Freiberg, has published some late researches on the nature of green stools, said to be of frequent occurrence in patients who are under a course of certain mineral waters. He altogether denies that any quantity of bile is present in the green evacuations of invalids and others who partook of the Carlsbad waters; but attributes the tint to the green sulphuret of iron, generated in the stomach and intestines by the reduction of the sulphate of soda of the water to a sulphuret, and its subsequent action on the iron held in solution in the water. He states, in confirmation of his view, that hydrochloric acid destroys the green color of the stools, evolving at the same time sulphuretted hydrogen. Dr. Frankl, however, of Marienbad, attributes the color of these evacuations to the "same source" as the green mucous discharges from the vagina in leucorrhœa, the urethra in gonorrhœa, and the nasal secretions in some forms of coryza.

Professor Lehmann calls attention to the green fluid or semi-fluid excrements which are not unfrequently discharged in typhus fever, even when no calomel has been administered, and adds: "bile-pigment and the biliary acids are only rarely to be detected in any quantity in such stools by a chemical investigation." I have observed the same character of discharges in the more malignant forms of scarlatina, and on submitting them to the test of the microscope have always found broken-up and distorted blood-corpuscles, together with an admixture of the elements of the blood, such as is seen in the green discharges of young children.

From long and close observation and careful analyses, Dr. Golding Bird arrived at the conclusion that green discharges have at least no *direct* connection with morbid biliary secretion, but that they are occasioned *by a morbid exudation from the intestinal mucous membrane.*

Believing that this view is in harmony with well-attested facts, I have publicly maintained it, for many years of my professional life, as probably the true pathology of these affections. It must be confessed, however, by those who maintain this view, that a morbid condition of the liver is often associated with these discharges. Indeed, so constant is this relation, that the older pathologists were led to attribute these discharges to a "morbid sympathy between the liver and intestines,"—an explanation which cannot be said to be remarkable for its clearness. Others, accommodating themselves to the more generally received opinion, and certainly to a more tangible pathology, attributed these discharges to an increased activity of the liver in throwing off "crude bilious matter;" whereas the true cause is referable (if the liver be at fault) not to sympathy, nor to excessive action of the liver, but to *congestion of the portal venous system.* Two conditions are associated with this as necessary concomitants: First, arrest of hepatic secretion, growing out of congestion; second, excessive acid fermentation of the fæcal contents, resulting from the absence of bile, as demonstrated by the experiments of Bidder, Schmidt, and others.

In infantile life, mucous membranes become active outlets for morbid action; and at no other period of life is there the same relative amount of lithic, lactic, and other acids formed. It is estimated by Dr. Rigby that a child eight years old eliminates as much urea as one of adult years. Hence, in cases of deranged assimilation, with arrest of hepatic secretion, the contents of the bowels are apt to become excessively acid, and this free acid, mingling with the elements of blood which slowly percolates through the over-strained capillaries of the mucous membrane, whose epithelium has been swept away, gives rise to frothy, green discharges, more or less mingled with fragments of blood-corpuscles, mucus-corpuscles, and the hæmatin of the blood.

In the examination of this subject, therefore, our attention should be mainly directed to the condition of the *mucous membrane* for an explanation of the phenomena of these discharges. An essential fact in their production *is the hyperæmic state of this tissue.* The result of this hyperæmia we will better understand by studying the process of "*exudations of mucous membranes,*" as described by Rokitansky. He points out the analogy of what he calls the "*croupy process,*" as occurring on *all* mucous membranes, by which the epithelium of the membrane is destroyed, and rapid exudation of plastic matter takes place upon its free surface. This exudative process gives rise, under certain conditions, "to a sanious exudation of a variously shaded brown or *green* color." At other times the products are either albuminous, jelly-like and pellucid, or quite colorless, as observed in Asiatic cholera.

The physiological habits of mucous membranes are quite peculiar. The epithelium covering the free surface is formed of minute scales, "packed in layers like figs in a box." The recent and inferior layer of cells projects against the superior and older cells. As secretion is increased by irritation of the mucous membrane, rapid desquamation of the epithelium takes place; in the pushing-out process the deeper layers throw off the superior cells before they have arrived at maturity; the nutritious matter cannot be consumed by those immature cells; and hence we find in the exudation, instead of healthy normal mucus, an increased quantity of immature epithelium, together with altered blood-corpuscles, more or less quantity of semi-fluid, muco-gelatinous matter, and substances which yield a cytoblastema for the higher development of cells.

This hyperæmia of the mucous membrane may readily extend, by continuity and similarity of structure, to the hepatic system, which, like the same structure in other regions of the body, is liable to all its varieties of inflammation.

Dr. Budd describes, among other varieties, what he calls, in accordance with the analogy of the same membrane in other organs, *croupal* or *plastic* inflammation, and suggests that this "bronchitis of the liver"

may play as important a part in diseases of the biliary apparatus as does bronchitis in those of the respiration.

It will readily be seen, by studying the law of inflammation of mucous membranes, that the character of the secretion or exudation on their free surface is an expression of the *degree of irritation*, rather than of the peculiar character of the inflammation. The same tissue is everywhere obedient to the same law of inflammation; and hence the interesting fact, that the microscope reveals, essentially, the same products of inflammation in the green and melænal discharges of childhood that have been observed in the *black vomit* of yellow fever. The difference is only a question of rapidity of exudation, and degree of inflammation. In either case a species of hemorrhage takes place from the congested or inflamed mucous surface. In the one instance the effusion is rapid and more copious, and the matter ejected is, therefore, more easily recognized as hemorrhagic in character; in the other, blood is exuded very slowly and in small quantities, giving rise to the dark-green stools characteristic of childhood,—the green color partly depending, it may be, upon the presence of bile-pigment.

Dr. Golding Bird has demonstrated, however, that green coloring matter may be generated in the animal economy from the action of certain matters on the hæmatosin or coloring matter of the blood, independently of the presence of bile-pigment. He cites the well-known fact, that when blood is exposed to the influence of sulphuretted hydrogen gas, it acquires a deep olive-green color when viewed by reflected light, and a dingy-red by transmitted light,—"phenomena identical with those presented by the coloring matter of the bile." He describes two products of the action of nitric acid upon a clot of blood—an olive-green sweetish substance, and an intensely bitter yellow one. To the former he applies the conventional term *chloro-hæmatin*, and to the latter that of *xanthe-hæmatin*. And, in the conclusion of a very able article upon the subject, adds:—

"Since, then, the coloring matter of the blood is fully capable of being converted into green pigments under the influence of different agents, it must, I think, be admitted that we are not to assume the green color of an animal excretion as of necessity depending upon an excess of bile. And when chemical analysis fails to indicate the presence of any quantity of this secretion in a bright-green evacuation, it is but legitimate to seek for some other cause of this tint. The proportions of the so-called biliverdine very closely approach to those of the xanthe-hæmatin before alluded to, and I confess that I am induced to regard the green color of the emerald and 'chopped spinach' stools of children as depending upon the presence of modified blood, rather than on an excess of bile.

"Believing that the green stools alluded to are but a form of melæna,

I have often closely questioned the nurses of children voiding them, regarding the appearance of the evacuations before and after the development of the green color, and have almost constantly been told that streaks, or even clots of blood, had been observed.

"I regard, then, the presence of green stools as indicative not of a copious secretion of bile, but of a congested state of the portal system, in which blood is exuded very slowly, and in small quantities, so as to allow the color to be affected by the gases and secretions present in the intestines; a state of things capable of readily ending in melæna, in which the effusion of blood is so copious and sudden as not to give time for the occurrence of the changes alluded to.

"There is, moreover, a peculiarity in the green dejections of children, and others whose portal circulation is congested, which, so far as I know, is quite distinct from any property presented by mere bile under similar circumstances. I allude to the effect of exposure to the oxygenating influence of the air upon them. When first voided, the 'chopped spinach' stools are, in the majority of cases, of a bright-orange color, and they assume their characteristic grass-green hue only after exposure to the air. The time required for this change varies remarkably. I have seen an orange-colored stool become green in a few minutes, and in the same patient, only a day or two afterwards, many hours may have been required to effect the same change."

To resume. In proof of the view here taken, that green and melænal discharges are not caused by the presence of an excess of bile, we have adduced the facts :—

1. That in a normal condition of the bowels the bile undergoes certain changes, and is absorbed on its road from the liver to the rectum.

2. When no bile flows into the intestine, rapid decomposition of the contents of the canal occurs, attended by putrid or sour discharges,—conditions characteristic of the green discharges of childhood.

3. The characteristic color of the fæcal dejections depends upon the presence of the *coloring matter* of the bile, not upon the presence of bile proper.

4. The green stools, *which follow the administration of calomel* depend upon the presence of the sulphide of mercury.

5. The analyses of Dr. Golding Bird, Simon, and others show but a *trace of bile* in such discharges.

6. No bile was discovered by Professor Kerstein in the green discharges of those who partook of the Carlsbad waters,—the color depending, as he supposed, upon the presence of the green sulphuret of iron.

7. The same green color is observed, under some circumstances, in the mucous discharges from the vagina in leucorrhœa, the urethra in gonorrhœa, in some forms of bronchitis, and in the nasal secretions in coryza.

8. Hyperæmia of the mucous membrane is often followed by rapid desquamation of its epithelium, and a consequent escape of the elements of the blood in such small quantities that its color is affected by the gases and secretions of the intestine.

9. In proof of this, the microscope reveals, in such discharges, altered blood-corpuscles, granules, free nuclei, and jelly-like fibro-albuminoid mucus, such as is observed in inflammatory exudations of mucous membranes.

10. The observations of Dr. Golding Bird demonstrate that green coloring matter *may* be generated in the animal economy from the action of certain matters on the hæmatosin, or coloring matter of the blood.

And, lastly, the fact that these discharges most generally occur in the warm summer and fall months is strongly confirmatory of the view here taken. The debilitating effects of heat, when combined with moisture, very readily develop diseases of the gastro-intestinal mucous membrane; for the reason that there is a direct relation, based on the general law of identity of structure, between the skin and mucous membrane of the bowels. "An important medical law," says Erasmus Wilson, "is founded on the continuity and similarity of structure of the investing and lining membrane of the body. This law resolves itself into three expressions—namely, *that disease affecting a part of a membrane is liable to spread to the whole; secondly, that disease of the mucous membrane may spread to the skin, and vice versa; and, thirdly, that disease of a part of a mucous membrane may be translated to a part of the skin, and vice versa.*"

This law of correlation between the cutaneous surface without and the mucous membranes within, has been long known and recognized. Thus, sudden changes from the extreme heat of mid-day to the damp chill of evening, morbidly impress the excitor or sensitive nerves of the skin, and, through the medium of the spinal centre, excite and modify the secretory function of the abdominal viscera. These suffer from congestion; their vital properties are lowered, and their functions consequently illy performed. The liver is readily involved in this morbid action. Between the extreme vessels of the *vena portæ* and the extreme vessels of the surface of the body, there exists one of the strongest sympathies in the human frame. The records of tropical climates, and of hot days and chilly nights in our own climate, furnish many striking illustrations of this law. And, for very obvious reasons, the liver becomes involved, in its vascular lesions, with other portions of the abdominal viscera. Congestion of the portal capillaries is followed by congestion of the veins and capillaries of the intestinal mucous membrane, and this condition, we can readily believe, may, under peculiar circumstances, be followed by percolation of more or less of the elements of the blood through the mucous coat of the intestinal canal.

In the foregoing paper I have endeavored to arrange and present some reliable facts and observations bearing upon a subject of acknowledged obscurity. If the views be correct, (and I claim for them well-attested observation,) they will very much modify the popular notion of the pathology and treatment of abdominal affections, characterized by green and melænal discharges.

